# Proinflammatory Interleukin-33 Induces Dichotomic Effects on Cell Proliferation in Normal Gastric Epithelium and Gastric Cancer

**DOI:** 10.3390/ijms22115792

**Published:** 2021-05-28

**Authors:** Laura Francesca Pisani, Gian Eugenio Tontini, Carmine Gentile, Beatrice Marinoni, Isabella Teani, Nicoletta Nandi, Pasquale Creo, Emanuele Asti, Luigi Bonavina, Maurizio Vecchi, Luca Pastorelli

**Affiliations:** 1 Gastroenterology ans Endoscopy Unit, IRCCS Policlinico San Donato, 20097 San Donato Milanese, Italy; laura.pisani@grupposandonato.it (L.F.P.); pasquale.creo@grupposandonato.it (P.C.); 2 Department of Biomedical Science for Health, Università degli Studi di Milano, 20133 Milano, Italy; gianeugenio.tontini@unimi.it (G.E.T.); carmine.gentile@unimi.it (C.G.); beatrice.marinoni@studenti.unimi.it (B.M.); isabella.teani@gmail.com (I.T.); nicoletta.nandi@studenti.unimi.it (N.N.); emanuele.asti@grupposandonato.it (E.A.); luigi.bonavina@unimi.it (L.B.); 3 Gastroenterology and Endoscopy Unit, Foundation IRCCS Ca’ Granda Ospedale Maggiore Policlinico, 20122 Milan, Italy; maurizio.vecchi@unimi.it; 4 Division of General Surgery, IRCCS Policlinico San Donato, 20097 San Donato Milanese, Italy; 5 Gastroenterology and Liver Unit, ASST Santi Paolo e Carlo, Ospedale San Paolo, 20100 Milano, Italy; 6 Department of Health Sciences, Università degli Studi di Milano, 20133 Milano, Italy; 7 Department of Pathophysiology and Trasplantation, Università degli Studi di Milano, 20133 Milano, Italy

**Keywords:** interleukin-33, gastric epithelium, proliferation, apoptosis, cell cycle, gastric cancer

## Abstract

Interleukin (IL)-33 is a member of the interleukin (IL)-1 family of cytokines linked to the development of inflammatory conditions and cancer in the gastrointestinal tract. This study is designed to investigate whether IL-33 has a direct effect on human gastric epithelial cells (GES-1), the human gastric adenocarcinoma cell line (AGS), and the gastric carcinoma cell line (NCI-N87) by assessing its role in the regulation of cell proliferation, migration, cell cycle, and apoptosis. Cell cycle regulation was also determined in ex vivo gastric cancer samples obtained during endoscopy and surgical procedures. Cell lines and tissue samples underwent stimulation with rhIL-33. Proliferation was assessed by XTT and CFSE assays, migration by wound healing assay, and apoptosis by caspase 3/7 activity assay and annexin V assay. Cell cycle was analyzed by means of propidium iodine assay, and gene expression regulation was assessed by RT-PCR profiling. We found that IL-33 has an antiproliferative and proapoptotic effect on cancer cell lines, and it can stimulate proliferation and reduce apoptosis in normal epithelial cell lines. These effects were also confirmed by the analysis of cell cycle gene expression, which showed a reduced expression of pro-proliferative genes in cancer cells, particularly in genes involved in G0/G1 and G2/M checkpoints. These results were confirmed by gene expression analysis on bioptic and surgical specimens. The aforementioned results indicate that IL-33 may be involved in cell proliferation in an environment- and cell-type-dependent manner.

## 1. Introduction

Gastric cancer is a leading cause of cancer-related deaths and is the fourth most common type of cancer in the world [1,2]. The overall survival rate of gastric cancer patients at 5 years is only about 10–30%. Major risks for the development of gastric cancer are *Helicobacter pylori* and Epstein–Barr virus infections, family history, dietary factors, alcohol consumption, smoking, and chronic gastritis [2,3]. 

The development of gastric cancer is a complex, multistep process involving multiple alterations in oncogenes, tumor suppressor genes, DNA repair genes, cell cycle regulators, and signaling molecules; thus, the pathways that lead to the transformation towards gastric cancer still need to be fully investigated [4,5]. Indeed, chronic inflammation plays a major role, and proinflammatory cytokines appear to promote progression from gastritis to cancer; in particular, the interleukin (IL)-1 family members, such as IL-1β and IL-18, have been shown to induce gastric carcinogenesis in animal models [6,7]. 

IL-33 has been identified as a new member of the IL-1 cytokine family and exerts its biological effects through the binding of its receptor, ST2, also known as IL-1 receptor-like 1 (IL1RL1), belonging to the Toll-IL-1 receptor (TIR) superfamily [6,8]. IL-33 appears to be a cytokine with dual function, acting both as a traditional cytokine and as an intracellular nuclear factor with transcriptional regulatory properties, and it is involved in gastrointestinal (GI) tract epithelial repair and restitution, promoting mucosal healing [9,10]. IL-33 is broadly expressed in many tissues, but its expression appears to be restricted by cell type [6]. Expression analysis of human and mouse cDNA libraries have revealed a high expression of IL-33 in barrier epithelia within organs/tissues in direct contact with the external environment, including skin, airway, and gut epithelia, suggesting a possible role of this cytokine in early immune responses against invasive pathogens [6]. At the cellular level, IL-33 is predominantly present in stromal cells, including fibroblasts, smooth muscle cells, and endothelial and epithelial cells [11], and in restricted populations of hematopoietic cells, such as macrophages [6,12].

Moreover, several studies have shown that normal mice injected with recombinant IL-33 develop marked epithelial cell hyperplasia throughout their whole gastrointestinal tract as well as features of spasmolytic polypeptide-expressing metaplasia in the stomach, with infiltration into the lamina propria of eosinophils and mononuclear cells and sustained chronic Th2-driven inflammation [13,14,15]. 

The role of IL-33 in neoplasia has been poorly investigated; however, different studies have reported both pro- and antitumorigenic functions [16]. It has been hypothesized that IL-33 has a protumorigenic function in cancer cell lines, inducing an increase in invasion, migration [4,17], and chemoresistance [18]. In vivo, the protein seems to be less abundant in gastric tumor tissues than in surrounding normal tissue, and it does not correlate to the patient’s prognosis [19]. These contrasting results suggest that the role of IL-33 in cancer development and growth must still be clarified. In order to unravel some of these aspects, we investigate whether IL-33 has effects on the cell cycle and apoptosis in in vitro and ex vivo settings.

## 2. Results 

### 2.1. IL-33 Is Expressed in Gastric Cell Lines and Its Expression Is Increased in Chronic Gastritis

**Figure 1:**
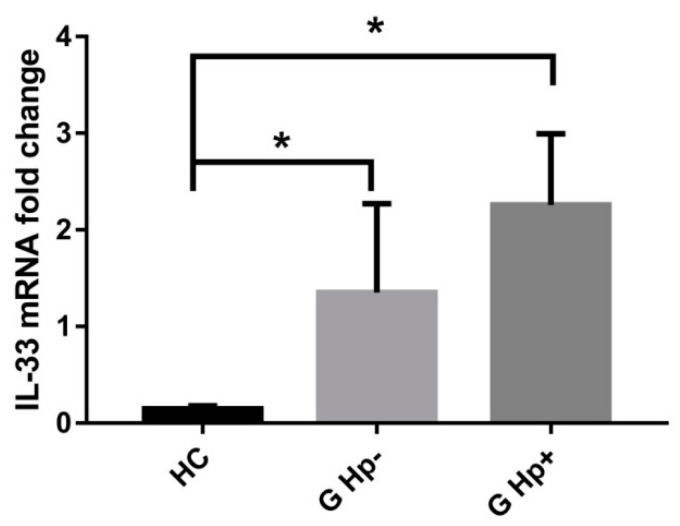
Gastric IL-33 is elevated in patients with a diagnosis of gastritis and even more in *H.pylori*-positive diagnosis. RealTime PCR in gastric biopsies showed a correlation between gastritis and *H.pylori*-positive gastritis. Data are shown as mean ± SD. HC: healthy controls; GHp−: gastritis *H.pylori*-negative; GHp+: gastritis *H.pylori*-positive. * *p* < 0.05.

By carrying out real-time PCR and Western blots, we have demonstrated the expression of IL-33 and its receptor ST2 in the GES-1, AGS, and NCI-N87 cell lines to validate our in vitro models (Figure S1). Given the fact that IL-33 was shown to be overexpressed during chronic inflammation, we evaluated its expression in gastric biopsies from patients who had undergone esophagogastroduedenoscopy (EGDS) procedures. Biopsies were collected and classified as normal tissue from patients without any gastric disease (HC; *N* = 48), gastritis *H.pylori*-negative (G Hp−; *N* = 21), and gastritis *H.pylori*-positive (G Hp+; *N* = 34) (Table 1). Data in Figure 1 show that IL-33 is overexpressed in gastritis *H.pylori*-negative samples (HC 0.15 ± 0.03 vs. G Hp− 1.75 ± 0.61; *p* < 0.05) and even more expressed in *H.pylori*-positive gastritis (HC 0.15 ± 0.03 vs. G Hp+ 2.26 ± 0.74; *p* < 0.05).

### 2.2. IL-33 Differentially Modulates Gastric Cell Proliferation and Migration

IL-33 treatment exerted opposite effects on the different cell lines considered. Gastric cancer AGS cell line proliferation was reduced after IL-33 exposure, while normal gastric GES-1 cell line proliferation was induced at the same experimental conditions.

**Figure 2:**
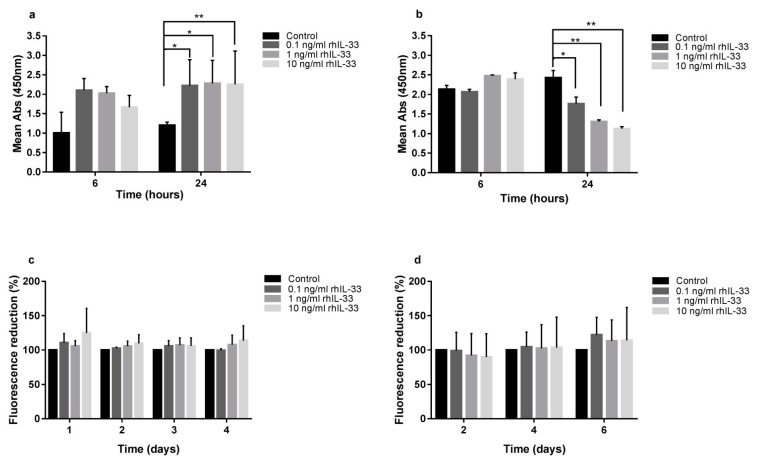
Exogenous administration of rhIL-33 induced the activation of a proliferative metabolic pathway in normal gastric epithelial cells, while the same challenge on neoplastic cells did not activate proliferation. Colorimetric XTT assay shows the proliferative effect on GES-1 (**a**) and the antiproliferative effect on AGS (**b**) after 24 h treatment; fluorescent CFSE assay (**b**–**d**) did not show any statically significant variation in proliferation. Data are shown as mean ± SD. * *p* < 0.05; ** *p* < 0.01.

In detail, the XTT proliferation assay on GES-1 cells showed an increase in proliferation after 24 h of treatment with rhIL-33 (ctrl 1.20 ± 0.08 vs. 0.1 ng/mL 2.61 ± 0.03, *p* < 0.05; ctrl 1.20 ± 0.08 vs.1 ng/mL 2.61 ± 0.25, *p* < 0.05; ctrl 1.20 ± 0.08 vs.10 ng/mL 2.75 ± 0.04, *p* < 0.01) (Figure 2a).

The CFSE assay showed a trend towards increased proliferation in IL-33-stimulated GES-1, with a dose–response correlation, but the data did not reach statistical significance (Figure 2c).

Conversely, the XTT proliferation assay on AGS cells showed a reduction of proliferation after 24 h of treatment with rhIL-33 (ctrl 2.43 ± 0.18 vs. 0.1 ng/mL 1.76 ± 0.17, *p* < 0.05; ctrl 2.43 ± 0.18 vs. 1 ng/mL 1.31 ± 0.04, *p* < 0.01; ctrl 2.43 ± 0.18 vs. 10 ng/mL 1.12 ± 0.05, *p* < 0.01) (Figure 2b).

Additionally, the CFSE assay showed no inhibitory effect of IL-33 on AGS (Figure 2d).

In order to assess whether or not the IL-33 inhibitory effect on cell replication was specific to the AGS cell line, we repeated the experiments on a different gastric cancer cell line, NCI-N87. With the XTT proliferation assay, the same inhibitory activity was observed, in which proliferation was reduced after 24 h of treatment (ctrl 2.70 ± 0.09 vs. 0.1 ng/mL 2.41 ± 0.01, *p* < 0.05; ctrl 2.70 ± 0.09 vs. 1 ng/mL 2.49 ± 0.043, *p* < 0.05; ctrl 2.70 ± 0.09 vs. 10 ng/mL 2.43 ± 0.01, *p* < 0.05) (Figure S2a), whereas the CFSE assay showed a dose-dependent reduced replication in NCI-N87 cells, which, however, was not statistically significant (Figure S1b).

We postulate that IL-33 can activate the metabolic pathways that will lead to the initiation of the cell cycle in GES-1 in the first 24-h challenge, whereas it induces opposing effects in AGS and NCI-N87 cell lines; furthermore, CFSE demonstrates that the IL-33 effect is maintained during the following days of culture.

**Figure 3:**
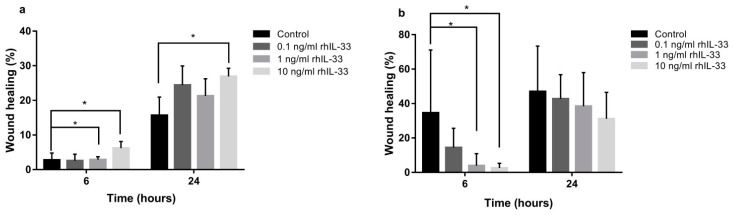
Administration of rhIL-33 for 6 and 24 h induced migration in the normal cell line (GES-1) (**a**), while migration was delayed in neoplastic cells (AGS) (**b**). Data are shown as mean ± SD. * *p* < 0.05.

The effect of IL-33 on migration in GES-1 was assessed by scratch assays, where wound closure was accelerated in a dose-dependent manner, with a concentration of 10 ng/mL at the 6-h (ctrl 2.76% ± 2.0 vs. 10 ng/mL 6.17% ± 1.9; *p* < 0.05) (Figure 3a) and 24-h timepoints (ctrl 15.67% ± 5.2 vs. 10 ng/mL 26.93% ± 2.35; *p* < 0.05) (Figure 3a). To assess that IL-33 could reduce AGS migration, wound closure was delayed when IL-33 was present, in a dose-dependent manner, starting from 6 h, with IL-33 concentrations of 1 and 10 ng/mL (ctrl 34.54% vs. 1 ng/mL 3.89%, *p* < 0.05; ctrl 34.54% vs. 10 ng/mL 2.55%, *p* < 0.05) (Figure 3b). Similarly, wound closure in NCI-N87 was delayed when 1 and 10 ng/mL of rhIL-33 was present after 24 h of treatment (Figure S3).

### 2.3. IL-33 Induces Apoptosis in Gastric Cancer Cell Lines, but Not in Non-Neoplastic Cells

**Figure 4:**
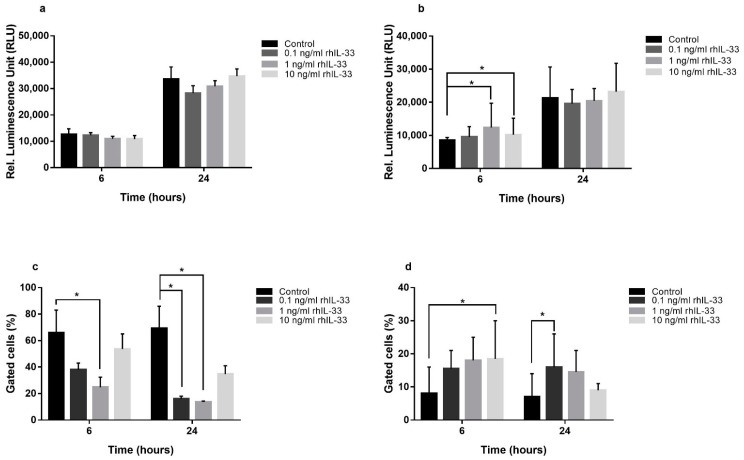
Exogenous treatment with rhIL-33 is able to modulate apoptosis. Quantitation of relative luminescence unit (RLU) for caspase 3/7 activity and the percentage of annexin-V-positive cells by cytofluorimetric assay show a reduction in early apoptosis in GES-1 (**c**) without variation in the activation of executioner caspases (**a**). In AGS, the same treatment increased both executioner caspases (**b**) and early apoptosis (**d**). Data are shown as mean ± SD. * *p* < 0.05.

Apoptosis is another parameter evaluated in this study. Our experiments showed that IL-33 had different effects on different cell lines. In GES-1, the evaluation of caspase-3 and caspase-7 activities did not show any variation in apoptosis (Figure 4a). The annexin V/propidium iodine assay showed a reduction in annexin V after 6 h (ctrl 66.0% ± 29.5 vs. 1 ng/mL 24.7% ± 13.2; *p* < 0.05) and 24 h of incubation with IL-33 (ctrl 69.0% ± 28.8 vs. 0.1 ng/mL 16% ± 3.5, *p* < 0.05, and ctrl 69.0% ± 28.8 vs. 1 ng/mL 13.7% ± 1.12, *p* < 0.05, respectively) (Figure 4c), indicating that IL-33 acts on GES-1 cells by reducing early events in apoptosis without the initiation of the extrinsic and intrinsic pathways of apoptosis, which end in the activation of executioner caspases.

In AGS, an evaluation of caspase-3 and caspase-7 activities showed an increase in apoptosis at 10 and 1 ng/mL of IL-33 after 6 h of treatment (ctrl 8510 ± 841 vs. 10 ng/mL 10,214 ± 496 and ctrl 8510 ± 841 vs. 1 ng/mL 12,338 ± 739; *p* < 0.05) (Figure 4b). These data were confirmed by the annexin V/propidium iodine assay, where IL-33 10 ng/mL increased annexin V after 6 h of treatment (ctrl 8.0% ± 11.3 vs. 10 ng/mL 18.5% ± 16.3; *p* < 0.05) and with IL-33 0.1 ng/mL after 24 h of treatment (ctrl 7.0% ± 9.0 vs. 0.1 ng/mL 16.0% ± 14.1; *p* < 0.05) (Figure 4d). 

Confirmatory experiments on NCI-N87, evaluating caspase-3 and caspase-7 activities, showed an increase in apoptosis after 24 h of treatment with 1 ng/mL ctrl (ctrl 24,938 ± 1537 vs. 1 ng/mL 35,442 ± 433; *p* < 0.05) (Figure S4a), while the annexin V/propidium iodine assay showed an increase in apoptosis at 0.1 ng/mL after 6 h of treatment (ctrl 13.4% ± 1.3 vs. 0.1 ng/mL 33.2% ± 2.9; *p* < 0.05) (Figure S4b), indicating that IL-33 could induce apoptosis in cancer cell lines.

### 2.4. IL-33 Acts on Cell Cycle Gene Expression in Gastric Cell Lines

**Figure 5:**
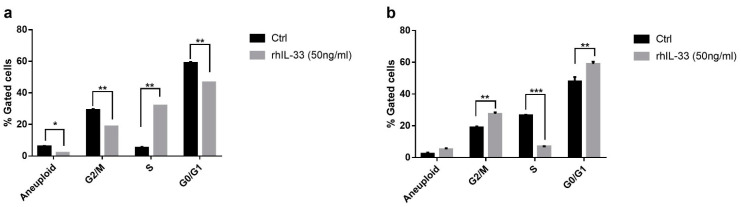
Cell cycle evaluation by staining of cells with propidium iodine after exogenous rhIL-33 treatment and cytofluorimetric cell count showed in (**a**) GES-1 an increase in the percentage of cells in phase S at the expense of the other two, perhaps for a block between S phase and G2 phase, while in (**b**) AGS, IL-33 increased the percentage of cells in phases G1/G0 and S, and the latter decreased considerably after 24 h treatment. Data are shown as the mean of % of gated cells. * *p* < 0.05; ** *p* < 0.01; *** *p* < 0.001.

In order to demonstrate the effect of IL-33 on AGS and GES-1 cell cycles, we conducted a series of cytofluorimetric analyses with propidium iodide staining after rhIL-33 stimulation. As shown in Figure 5a, in GES-1, IL-33 induced an increase in phase S at the expense of the other two, perhaps for a block between the S phase and the G2 phase. In particular, nonstimulated GES-1 vs. rhIL-33-treated cells exhibited a percentage of G0/G1 transition cells of 59.4% vs. 47.5% (*p* < 0.01); in phase S, 5.7% vs. 31.3% (*p* < 0.01); and in the G2/M transition, 28.7% vs. 17.8% (*p* < 0.01). In AGS, rhIL-33 seems to have the opposite effect; in fact, there is a block between phases G1/G0 and S, and the latter decreased considerably after treatment at 24 h, which means that the cells remained in the G1 phase. In particular, nonstimulated AGS vs. rhIL-33-treated cells exhibited a percentage of G0/G1 transition cells of 49.9% vs. 59.9% (*p* < 0.01); in phase S, 26.9% vs. 7.1% (*p* < 0.001); and in the G2/M transition, 19.4% vs. 26.7% (*p* < 0.01) (Figure 5b).

To further explore the potential molecular mechanisms underlying IL-33-mediated cell proliferation changes, we studied the expression of the cell cycle. We compared GES-1 cells with AGS tumor cells, reporting the differential expression of six genes (Table 2). In particular, cyclin C (CCNC), whose activation controls the G0/G1 transition, and cyclin E1 (CCNE1), whose activation promotes the G1/S transition, showed an increase of 6.11 and 6.04 times, respectively, in AGS vs. GES-1 cells (*p* < 0.05). Cellular regulatory genes such as BRCA and CDKN1 A interacting protein (BCCIP), cyclin B1 (CCNB1), and Karyopherin 2 (KPNA2) genes, which are involved in the G2/M transition and DNA damage and repair control points, increased by 2.34, 2.39, and 3.05 times, respectively, in AGS vs. GES-1 cells (*p* < 0.05). The expression of caspase 3 (CASP3) was increased by 24.08 times (*p* < 0.05) in AGS vs. GES-1 cells. Afterward, cells were challenged for 24 h with rhIL-33 (10 ng/mL), and we observed that in AGS, the stimulation with rhIL-33 decreased the expression of the selected genes. In particular, CCNC and CCNE1 showed a decrease of 3.31 and 4.15 times, respectively (*p* < 0.001), and BCCIP and CCNB1 decreased by 1.65 times (*p* < 0.05). The expression of CASP3 was more than halved by the treatment by 10.57 times (*p* < 0.05), suggesting that IL-33 was promoting the initiation of the apoptotic cascade through the induction of caspase synthesis. In GES-1, the stimulation increased the expression of all the six genes considered, but only KPNA1 reached a statistically significant modulation in comparison to untreated GES-1 cells (1.46-fold, *p* < 0.05). 

### 2.5. IL-33-Stimulation of Human Gastric Biopsies and Surgical Specimens Modifies Cell Cycle Gene Expression

**Figure 6:**
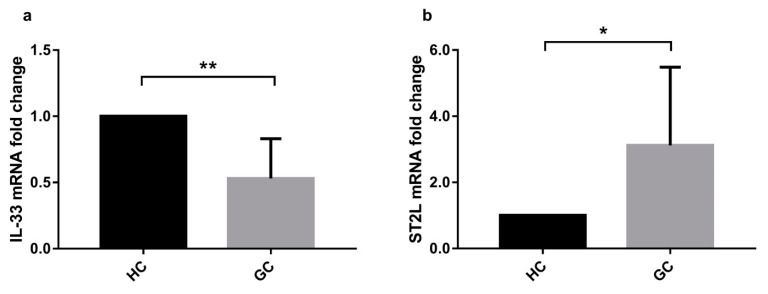
Gastric IL-33 and its receptor ST2 are modulated in gastric biopsies. mRNA relative expression was determined by Real-time PCR on gastric biopsies and showed that (**a**) IL-33 was downregulated in gastric cancer biopsies vs. normal gastric biopsies, while (**b**) its receptor ST2 was overexpressed in gastric cancer biopsies vs. healthy controls. Data are shown as mRNA fold change ± SD. HC: healthy control; GC: gastric cancer biopsies. * *p* < 0.05; ** *p* < 0.001.

Before testing the cell cycle gene expression after the treatment with rhIL-33 on ex vivo samples, we checked the expression of IL-33 and its receptor ST2 on biopsies collected from patients who had undergone endoscopy and were classified as healthy control (HC; *N* = 48) and gastric cancer patients (GC; *N* = 16) (Table 3). Results showed that IL-33 was downregulated in the GC group vs. HC (0.53 ± 0.30; *p* < 0.001) (Figure 6a), while ST2 L was upregulated in gastric cancer patients (3.12 ± 2.30; *p* < 0.05) (Figure 6b). 

**Figure 7:**
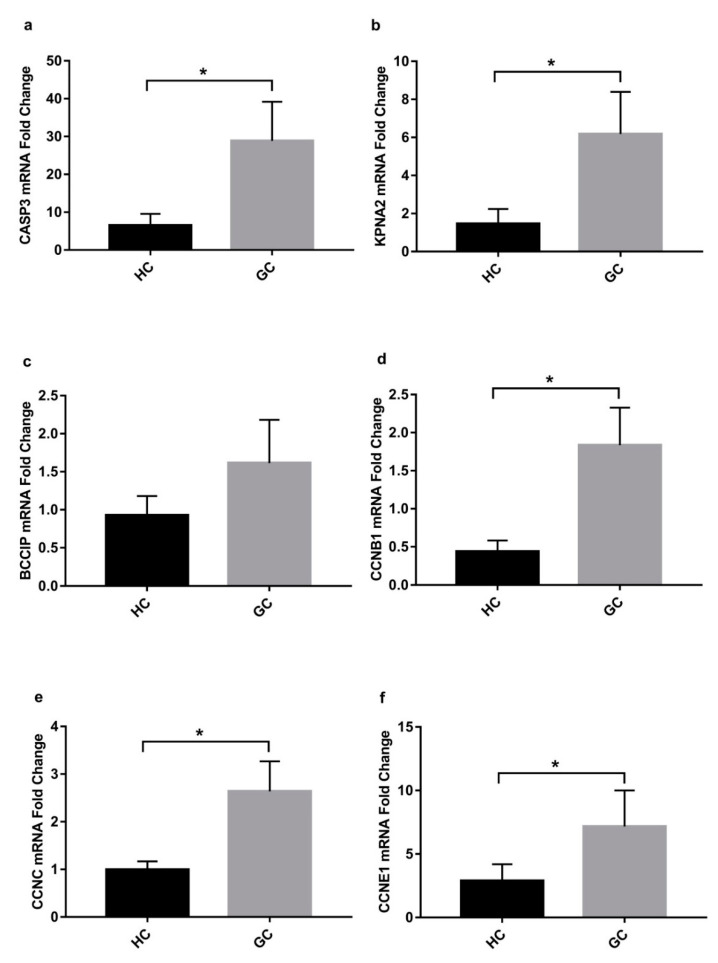
Real-time analysis of cell cycle genes performed on biopsies of gastric cancer tissue shows that the genes differentially modulated in cell lines are overexpressed in gastric cancer in comparison to healthy controls (**a**–**f**). Data are shown as mRNA fold change ± SD. HC: healthy control; GC: gastric cancer biopsies. * *p* < 0.05.

To further explore the potential molecular effect of IL-33 on ex vivo human gastric tissues, we first determined the expression by RT-PCR of the six genes identified in the cell line profiler in biopsies from normal gastric tissue and cancer tissue coming from different patients (Table 1). Figure 6 shows that all the genes modulated in cell lines were overexpressed in the gastric cancer samples (*p* < 0.05). Only BCCIP, even if overexpressed, did not reach statistical significance (Figure 7c). 

**Figure 8:**
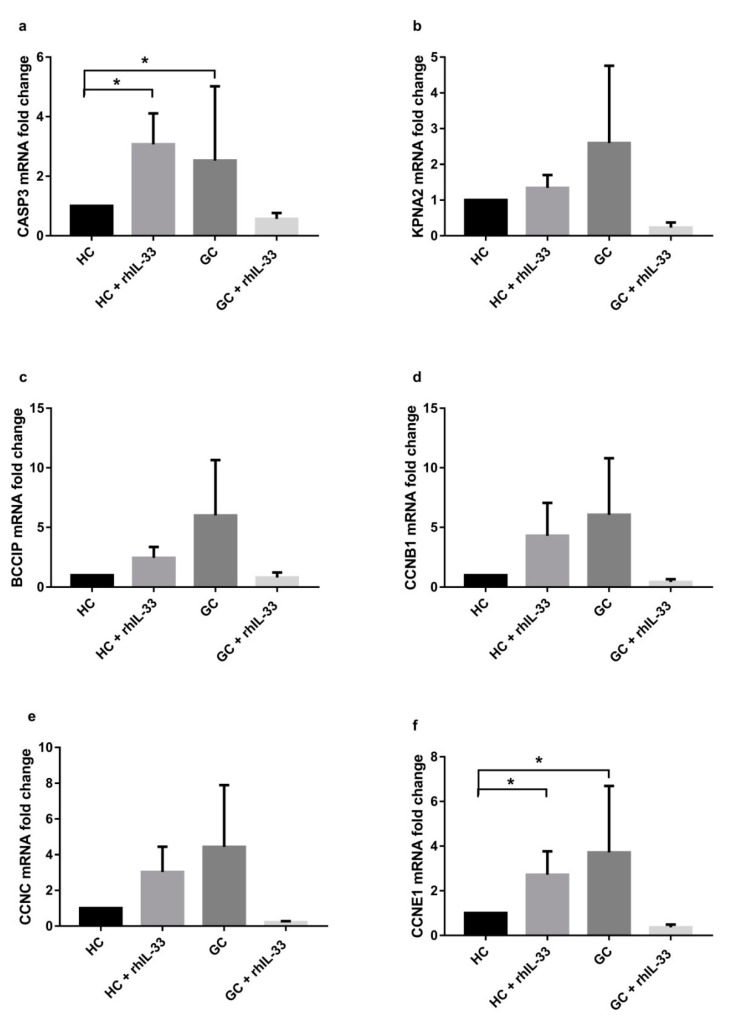
Cell cycle gene expression evaluated by RT-PCR in surgical specimens from gastric cancer patients after in vitro short-term culture, with or without rhIL-33, for 48 h shows that they can be modulated in both HC and gastric cancer samples (**a**–**f**). For each gene, IL-33 treatment can induce the expression in HC, while it is downregulated in GC. Data are shown as mRNA fold change ± SD. HC: healthy control; HC + rhIL-33: healthy control treated with rhIL-33; GC: gastric cancer tissue; GC + rhIL-33: gastric cancer tissue treated with rhIL-33. * *p* < 0.05.

Subsequently, we performed the same analysis on surgical specimens from three patients (mean age 75 ± 11) with gastric cancer after stimulation with rhIL-33 (10 ng/mL) for 48 h. Samples defined as healthy control (HC) and gastric cancer (GC) came from the same patient, identifying with HC in a region of the stomach far from the region with confirmed gastric cancer. Figure 8 shows that all genes were overexpressed in the GC samples vs. HC tissue. In particular, CASP3 after treatment with rhIL-33 was upregulated in HC (3.07 ± 1.04 vs. ctrl; *p* < 0.05) and downregulated in the GC samples (0.57 ± 0.20 vs. ctrl; *p* < 0.05), showing an effect on induction of apoptosis ex vivo (Figure 8a). All the other gene expressions were downregulated in the GC samples after treatment with rhIL-33 (Figure 8b–f), suggesting a role of this cytokine in the reduction of neoplastic cell proliferation. In normal tissue, CCNC and CCNE1 were upregulated after treatment with rhIL-33, suggesting a role in the induction of the transition of cells through G0/G1 and G1/S checkpoints (Figure 8e,f).

## 3. Discussion

The persistence of long-standing chronic inflammation is known to be of paramount importance in the development of gastric cancer; the precise role of each molecule participating in the proinflammatory milieu in chronic gastritis has yet to be fully investigated. Herein, we confirm that IL-33 is highly expressed in gastric mucosa during gastritis, and we have identified contrasting effects of this molecule on normal and neoplastic gastric epithelium. In fact, the incubation of GES-1 with rhIL-33 resulted in increased cell proliferation and migration, with no changes in apoptosis rates. These results are consistent with the observations that have shown that exposure to IL-33 causes histological changes in the lungs and GI tract, maintaining the turnover of the intestinal mucosa [20], intestinal homeostasis [21], and barrier function [9], including epithelial cell hyperplasia and hypertrophy [6] and also metaplasia [5,15,22]. These results also reveal a potential role of IL-33 in the epithelial alterations preceding the neoplastic transformation.

This effect on the proliferation of epithelial cells might be determined by the direct action of IL-33 on the epithelial cells, given the expression of the ST2 receptor on the proliferating epithelium. Unlike GES-1, the administration of rhIL-33 to AGS tumor cells led to a reduction in proliferation, which was accompanied by an intensification of apoptosis. Overall, these results are of great interest, as they propose a direct antiproliferative action of the cytokine.

It is not the first time that IL-33 has been demonstrated to have antitumor properties; however, previous studies have suggested that these effects could be mediated mostly by the influence of cytokines on immune system activation [23]. Moreover, while the stimulation of cell proliferation has been demonstrated on different immune [24], stromal [25], and epithelial [26] cell lines, the antiproliferative effect of IL-33 is relatively new in the literature. It has been found only in two cases: on a non-neoplastic murine fibroblastic cell line in a study conducted by Tominaga et al. [27], and, more recently, in neoplastic pancreatic cells in a recent study by Fang et al. [28], in which the authors found that IL-33 is able to suppress cell proliferation only in quiescent cells, while it stimulates replication in proliferating cells. IL-33 appears to exert its effect only on cells in the G0 phase, suggesting that the cytokine acts through a block between the G0/G1 phase, which could be mediated by the increase in cell cycle inhibitory proteins [29]. These observations are partially transferable to the cell lines used in our study. In fact, it can be speculated that IL-33 acts with antiproliferative and proapoptotic effects only on the quiescent AGS, thus determining a significant reduction in replication, which, however, remains higher than that of normal GES-1. Our cytofluorimetric analysis of the cell cycle is consistent with the hypothesis of a block in the G0/G1 transition. This analysis demonstrates a significant reduction in the percentage of cells in the S phase in AGS, suggesting a block upstream of the S phase, such as a block in G0/G1. Furthermore, the reduction of the expression of the cyclin C (CCNC) gene, together with cyclin-dependent kinase 3 (Cdk3), is necessary for the entry of quiescent cells into the G1 phase [30] and could be responsible for this effect. CCNC, in fact, forms a complex with the Rb protein and Cdk3, triggering its enzymatic activity, which is sufficient for the cell to re-enter the cell cycle [31]. We focused on additional genes that showed variations in opposite directions in normal and neoplastic cells, reflecting the contrasting effects of IL-33.

**Figure 9:**
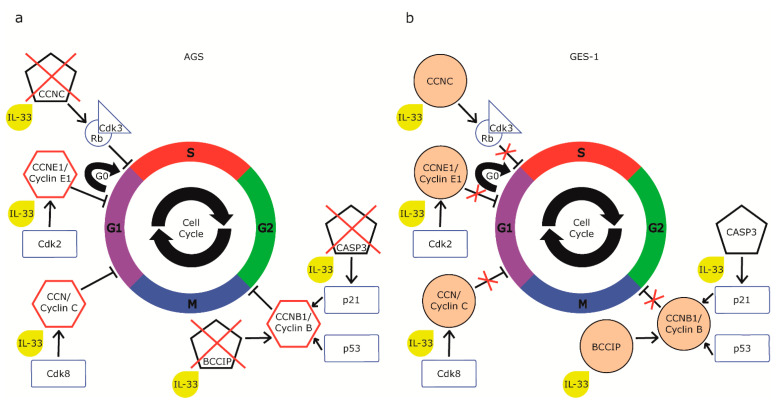
Schematic representation of cell cycle genes modulated by IL-33 shows that (**a**) in AGS, IL-33 interacts with genes that block G0/G1 transition as CCNC and G2/M transition as BCCIP and CASP3, leading to a reduction of cell proliferation, while (**b**) the induction of the overexpression of the same genes in GES-1 leads to an increase in proliferation.

In AGS cells, at baseline, these genes were expressed at much higher levels than in GES-1, as can be expected in tumor cells; however, treatment with rhIL-33 caused a significant decrease in the expression of each of them. This is consistent with the antiproliferative effect we observed on these cells. Instead, the reduction of CASP3 expression in AGS has an interesting implication on the effects on the cell cycle. In fact, caspase 3 is also capable of cleaving the p21 protein, a repressor of progression in the cell cycle, which acts on stimulation of the p53 protein, degrading cyclin B, which is responsible for the G2/M transition. The same activity is performed by the BCCIP gene product, which is also downregulated in AGS. Consequently, the reduction of the expression of CASP3 and BCCIP would also result in the reduced degradation of p21 and, consequently, greater inhibition of proliferation (Figure 9). Consistently, our results showed the reduction of the mRNA of CCNB1, coding for cyclin B, a target of p21. Data obtained on pancreatic cancer cells further support our results. In fact, pancreatic neoplastic cells showed an increased expression of p53, p15, and p21, all of which are cell proliferation inhibitors [28]. On the other hand, the expression of cell-cycle-progression-promoting genes, Cdk4 and Cdk2, decreased in pancreatic cells incubated with IL-33 [28]. Cdk2 is the kinase that interacts with cyclin E, also downregulated in AGS, while Cdk4 binds to cyclin D, forming a further complex that is involved in the first phase of G1 and is a potential site of the block of the S phase in AGS (Figure 9). All of these results have been proven to be coherent and complementary with ours and deserve further discussion.

At this stage, further investigation into the downstream signaling pathway activated in tumor cell lines is warranted in order to understand the exact mechanism by which IL-33 promotes apoptosis and inhibits proliferation. Nonetheless, we cannot exclude that the exogenous administration of IL-33 may have some off-target effects and be toxic to cells. Furthermore, several recent reports have supported an antitumorigenic role of the IL-33/ST2 axis in colon cancer. In particular, IL-33 has been shown to promote the function of CD8+ T-cells and NL cells, thus promoting tumor eradication [23,32]. Additionally, the expression of ST2 L was found to be lower in human tumors relative to adjacent non-neoplastic tissue—the higher the tumor grade, the lower the expression of ST2 L. All together, these results support a potential antitumorigenic role of the IL-33/ST2 axis.

After having verified the expression of IL-33 and its receptor in gastric biopsies from healthy and gastric cancer patients, we evaluated the expression of the cell cycle candidate genes before and after stimulation with the cytokine and, as Kania et al. [33] and Sun et al. [34] reported, we observed that CASP3 expression was decreased in patients with gastric tumors compared to normal gastric tissue. The prognostic value of some proteins of the CASP family in gastric cancer (GC) and, in particular, of CASP3 was associated with favorable clinicopathological features and a positive prognosis after curative surgery [35]. The 5-year overall survival rate of gastric cancer patients with a higher expression of CASP3 is 51.2% [36]. Considering these results, caspase-3 may act as a tumor suppressor in human gastric cancer, and the data obtained by our ex vivo experiments show that the effect of IL-33 on the induction of CASP3 supports the potential role of IL-33 as an antitumor factor. In gastric cancer, information on DNA copy number changes and gene expression changes showed differential gene expression between gastric tumor tissues and normal gastric tissues; the main genes are involved in basic functions such as the cell cycle, transcription, metabolism, signal transduction, and DNA replication. CCNC is among the genes with reduced copy numbers and mRNA expression in gastric cancer vs. normal tissue [37]. It has been reported that CCNC is deleted in patients with acute lymphoblastic leukemia [38], supporting the possibility that the *CCNC* gene is closely linked to tumor suppressor genes. Our data show that CCNC is downregulated in gastric cancer and that the treatment with rhIL-33 further reduces its expression. 

## 4. Materials and Methods

### 4.1. Cell Lines and Treatments

Human gastric epithelial cells (GES-1; courtesy of Prof. Hong Cai Beijing Cancer Hospital, Beijing, China) were cultured in RPMI-1640 (Sigma-Aldrich, Milan, Italy) supplemented with 10% fetal bovine serum (FBS), 1% antibiotic solution (100 U/mL penicillin, 100 mg/mL streptomycin) at 37 °C in a 5% CO_2_ humidified atmosphere. Gastric adenocarcinoma cell line AGS (ATCC^®^ CRL-1739™) was cultured in F12 nutrient mix, Kaighn’s Modification (Life Technology, Monza, Italy). NCI←−N87 [N87] (ATCC^®^ CRL-5822™) was cultured in RPMI-1640 medium (Sigma-Aldrich, Milan, Italy) supplemented with 10% FBS and 1% antibiotic solution (100 U/mL penicillin, 100 mg/mL streptomycin) at 37 °C in a 5% CO_2_ humidified atmosphere. The NCI-N87 cell line was used in this paper to confirm the data obtained in AGS and shed light on the not-cell specific effect of IL-33.

Cells were plated at 2.5 × 10^5^/mL; after 2 days, the cells reached 70% confluence and were used for the treatments. Cells were challenged with growing concentrations of recombinant human IL-33 (Alexis, Vinci Biochem, Vinci, Italy) at 37 °C for the incubation times indicated in the figure legends; rhIL-33 were suspended in sterile water and then diluted in complete medium specific to each cell line and added to cells to reach different final concentrations (0.1, 1, and 10 ng/mL). In the control groups, cells were incubated with the same amount of complete medium without rhIL-33.

### 4.2. Wound Healing

GES-1, AGS, and NCI-N87 were grown to confluence in µ-Dish 35 mm, low (Ibidi, Gräfelfing, Germany). The biocompatible silicone insert allows cells to grow in two separate chambers, making a gap of 500 ± 50 µm. Medium was replaced by complete medium and medium with rhIL-33 (0 h time point). Images were taken at 0, 6, and 24 h using JuLI™ Stage Live-Cell Imaging (PerkinElmer, Milan, Italy). Cell migration distance was determined by subtracting values obtained at 0 h from each time point. Migration distances are expressed as percentages over control values.

### 4.3. Evaluation of Cell Proliferation

Cell proliferation was evaluated using an XTT Cell Proliferation Assay Kit (ATCC, LGC Standards S.r.l., Sesto San Giovanni, Italy) on 5 × 10^5^ cells/well. Cells were seeded in 96-well plates in triplicates in 100 μL of complete medium 18 h before stimulation. After that, different concentrations of rhIL-33 were added to cells and incubated for 6 and 24 h. Activated XTT solution was prepared by adding 100 μL of the activation reagent to 5.0 mL of the XTT reagent. Then, 50 μL of the activated XTT solution was added to each well, and the plate was returned to the CO_2_ incubator for the optimized assay incubation time. Formazan formation was evaluated, recording absorbance at 450 nm wavelength using a microplate reader (Victor 3; PerkinElmer, Milan, Italy), and expressed as mean absorbance ± SEM. 

Cell proliferation rate was also determined using flow cytometric analysis with a CellTrace™ CFSE Cell Proliferation Kit (Life Technologies, Milan, Italy). Cells were harvested by trypsin digestion, washed three times, and resuspended in 2 mL PBS. Four microliters of CFSE stock solution were applied to cells to get a final concentration of 10 µM. Cells were incubated for 10 min at 37 °C. The staining was quenched by the addition of 5-volumes of ice-cold complete medium and incubated for 5 min in ice. Cells were washed two times and resuspended in complete medium with different rhIL-33 concentrations and further cultured for up to 4, 6, or 9 days.

XTT and CFSE assays were performed in triplicate, starting from three separately initiated cultures.

### 4.4. Cell Cycle Analysis by Propidium Iodine (PI) Staining

Cells were trypsinized and washed once in PBS to remove residual serum and trypsin and then resuspended in cold 70% ethanol, drop by drop, while vortexing to the number of 1.5 × 10^6^, and fixed for 30 min in ice. Cells were washed once in PBS, spinned at 850 g, 5 min, 4 °C and resuspend in PBS added with 1% bovine serum albumin (BSA), spinned at 850× *g*, 5 min, 4 °C and resuspend in 500 µL of PBS with 1% BSA and 0.1% Tween.

Finally, they were incubated for 15 min with RNAse A, followed by the addition of 10 µl of propidium iodine. Cell cycle analysis was performed by flow cytometry in triplicate (Perkin Elmer, Milan, Italy).

### 4.5. Analysis of Cell Cycle by RT^2^ Profiler PCR Array for Cell Cycle

The Human Cell Cycle RT2 Profiler PCR Array (PAHS-0820, SABiosciences, Qiagen, Italy) was used to evaluate the expression of 84 specific genes related to the cell cycle. After 24 h of culture with rhIL-33 (10 ng/mL) and without any challenge, total RNA was isolated using the miRNeasy Mini Kit (Qiagen, Milan, Italy) according to the manufacturer’s instructions. cDNA was synthesized from 500 ng of the total RNA using the RT2 First Strand Kit (Qiagen, Milan, Italy). PCR arrays were performed in 96-well plates on a StepOne Plus instrument (Life Technology, Monza, Italy). Briefly, the reaction mix was prepared from 2 × SABiosciences RT2 qPCR Master Mix and 102 µL of sample cDNA. Then, 10 µL of this mixture was added to each well of the PCR array. The thresholds and baselines were set according to the manufacturer’s instructions, and data were analyzed using software supplied by Qiagen (http://www.sabiosciences.com/pcr/arrayanalysis.php, accessed on 23 November 2020). The fold change in gene expression was calculated using the ΔΔCt method. More than 1.5-fold change and *p* < 0.05 in gene expression compared to unstimulated control were considered the up- or dowregulation of a specific gene expression. The gene pathway modified by IL-33 was reconstructed by IPA Software (SABiosciences, Qiagen, Milan, Italy). The genes differentially expressed in the two cell lines were tested by qRT-PCR after total RNA extraction from cells under various culture conditions using the RNeasy Kit (Qiagen, Milan, Italy), following the manufacturer’s instructions.

### 4.6. Evaluation of Caspase-3 and Caspase-7 Activities

Following 6 and 24 h treatments, cells were subjected to caspase 3/7 activity measurement with the Caspase-Glo assay kit (Promega, Milan, Italy). Briefly, the plates containing cells were removed from the incubator and allowed to equilibrate to room temperature for 30 min. Then, 100 μL of Caspase-Glo reagent was added to each well, and the content of the well was gently mixed with a plate shaker at 400 rpm for 30 s. The plate was then incubated at room temperature for 2 h. The luminescence of each sample was measured in a plate-reading luminometer (Victor 3; PerkinElmer, Milan, Italy), with parameters of 1 minute lag time and 0.5 second/well read time. The experiments were performed in triplicate and repeated on three separately initiated cultures.

### 4.7. Annexin V Apoptosis Detection

Cells were treated for 6 and 24 h, and, at each time point, they were subjected to an Annexin V-FITC Apoptosis Detection Kit (Enzo Life Science—Euroclone S.p.A, Milan, Italy). After each treatment, 5 × 10^5^ cells/mL were washed in PBS and resuspended in 195 µL of binding buffer added with 5 µL of annexin V-FITC and incubated for 10 min at room temperature. Afterward, cells were washed twice with PBS and resuspended in 190 µL of binding buffer, adding 10 µL of propidium iodide (20 µg/mL). The number of apoptotic cells was determined by flow cytometer (PerkinElmer, Milan, Italy).

### 4.8. Gastric Tissue Sample

Human gastric tissue was collected by Endoscopy Unit staff and by the Surgery Unit at IRCCS Policlinico San Donato, Italy. Endoscopists provided biopsy samples of gastric mucosa that were classified as normal tissue from patients without any gastric disease, gastritis *Helicobacter-pylori*-negative, gastritis *Helicobacter-pylori*-positive at histology, and gastric adenocarcinoma after evaluation by a pathologist. Biopsies were immediately frozen in liquid nitrogen for further analysis. Surgical samples were collected from patients undergoing surgical procedures for gastric cancer removal. Full-thickness samples were collected from the same patient after resection from the neoplastic area defined as gastric cancer (GC) and the non-neoplastic region of the stomach, far from the region with confirmed gastric cancer, defined as healthy control (HC). Specimens were placed in cold PBS with 1% antibiotic solution (100 U/mL penicillin, 100 mg/mL streptomycin). Samples were washed in PBS, cut in 0.5 cm^3^ fragments, and cultured in Dulbecco’s modified Eagle’s medium (DMEM), supplemented with 10% FBS, 1% antibiotic solution (100 U/mL penicillin, 100 mg/mL streptomycin) at 37 °C in a 5% CO_2_ humidified atmosphere. Samples from cancer and healthy tissue were randomly assigned to be challenged with rhIL-33 (10 ng/mL) or without challenge for 48 h. After the treatment, the samples were frozen in liquid nitrogen for further analysis. Total RNA was isolated from tissue samples by means of the TRIzol^®^ reagent (Life Technologies), following the manufacturer’s instructions.

### 4.9. Reverse Transcription and Real-Time PCR

Briefly, 1 µg of total RNA from the cells or tissues was subjected to DNase treatment (ThermoScientific, Monza, Italy) and then reverse-transcribed using Oligo (dT)_18_ primers and a RevertAid H Minus First Strand cDNA Synthesis Kit (ThermoScientific, Monza, Italy). These cDNA preparations were subjected to quantitative real time-PCR using Maxima SYBR Green qPCR Master Mix (ThermoScientific, Monza, Italy) and specific target gene primers (Table S1), normalized to human glyceraldehyde 3-phosphate dehydrogenase (GAPDH) and ribosomal protein S14 and reported as relative-fold-change among the groups, with baseline control set at 1.

### 4.10. Statistical Analysis

Statistical analysis was performed by GraphPad Prism 7 (GraphPad Software Inc., San Diego, CA, USA) using the appropriate statistical tests according to the underlying distribution of data, with *p* < 0.05., *p* < 0.01, and *p* < 0.001 considered statistically significant. 

### 4.11. Ethical Committee and Informed Consent Statement

The research was carried out according to The Code of Ethics of the World Medical Association and the Declaration of Helsinki. The study protocol was approved by the local Ethics Committee of ASL Milano 2 (protocol n.2725–2012). All patients enrolled were informed of the study, and written informed consent for the research use of the samples was obtained before endoscopy or surgery.

## 5. Conclusions

These results, which may seem puzzling, further confirm that the role of IL-33/ST2 in the progression from precancerous lesions to cancer, which has yet to be fully understood. Along these lines, recent studies investigating the role of IL-33/ST2 in colon cancer have reported contrasting results. In fact, while some studies have reported the downregulation of IL-33 in neoplastic tissues [39], as in our study, Maywald et al. (2015) reported the opposite [40] and suggested a pathogenic role of IL-33 in the development of cancer in a murine model of familial adenomatous polyposis. However, since IL-33 is known to be released upon cell damage, it is plausible to speculate that the chemotherapeutic regimes directly affected the levels of IL-33 in these patients [39].

Thus, while targeting IL-33 could represent a novel therapeutic approach to stop the progression of gastritis to cancer in the early phases of tumorigenesis, at this stage, we cannot exclude that IL-33 may have a role in antitumoral activity in the advanced stage of gastric cancer. It could, thus, respond to the urgency of identifying new and more effective weapons against this neoplasm. In fact, in Western countries, the decrease in gastric adenocarcinoma mortality observed recently depends more on the reduction of incidence than on the improvement of treatment. Because of the difficulty of implementing screening programs in the Western world, gastric cancer is mainly diagnosed in the advanced stage, resulting in a negative prognosis [41]. However, we must not forget that the opposite, stimulating effects on proliferation, observed on gastric epithelial cells, could mean a pro-proliferative effect on the normal epithelium. Consequently, it is important to further investigate the molecular pathways involved in order to identify the correct targets for possible therapeutic manipulation of the IL-33/ST2 axis. Further studies are required before any definitive conclusion can be made.

## Figures and Tables

**Table 1:** Demographic of patients enrolled for biopsy specimen collection.

Group	*N*	Gender	*n* (%)	Age (Mean ± SD)
Healthy Controls (HC)	48	F M	33 (68.8) 15 (31.3)	60.7 ± 15.1
Gastritis *H.pylori*-negative (G Hp−)	21	F M	10 (47.6) 11 (52.4)	71.5 ± 14.1
Gastritis *H.pylori*-positive (G Hp+)	34	F M	21 (61.8) 13 (38.2)	66.2 ± 18.5

**Table 2:** Cell cycle genes differentially regulated in GES-1 and AGS cells after rhIL-33 stimulation. Using GES-1 as control cell line, we selected a subset of genes that were differentially regulated between the two cell lines.

GeneSymbol	GES-1 (Fold Change)	GES-1 + rhIL-33 (Fold Change)	*p*-Value	AGS (Fold Change)	*p*-Value	AGS + rhIL-33 (Fold Change)	*p*-Value
BCCIP	1.000	1.453	0.281	2.395	0.002	1.649	0.019
CASP3	1.000	1.818	0.141	24.084	0.00003	10.566	0.010
CCNB1	1.000	1.446	0.284	2.389	0.002	1.653	0.019
CCNC	1.000	1.473	0.265	6.105	0.053	3.313	0.0004
CCNE1	1.000	1.153	0.576	6.035	0.0001	4.145	0.0001
KPNA2	1.000	1.456	0.006	3.046	0.0002	1.039	0.744

**Table 3:** Demographic of patients enrolled for bioptic specimen collection for the evaluation of cell cycle gene expression.

Group	*N*	Gender	*n* (%)	Age (Mean ± SD)
Healthy Controls (HC)	48	F M	33 (68.8) 15 (31.3)	60.7 ± 15.1
Gastric cancer (GC)	16	F M	10 (62.5) 6 (37.5)	75.6 ± 8.1

## Data Availability

The data presented in this study are available on request from the corresponding author.

## Supplementary Materials

The following are available online at https://www.mdpi.com/article/10.3390/ijms22115792/s1, Figure S1: IL-33 and its receptor ST2 proteins, detected (a) by Western blot in the three cell lines and mRNA expression (b) by real-time PCR, show that IL-33 and ST2 L were present in GES-1, AGS, and NCI-N87 cell lines, Figure S2: Exogenous administration of rhIL-33 reduced the activation of the proliferative metabolic pathway in neoplastic gastric epithelial cells, while the same challenge on neoplastic cells did not activate proliferation, Figure S3: Administration of rhIL-33 for 6 and 24 h migration delayed the wound closure in neoplastic cells NCI-N87 after 24 h treatment, Figure S4: Exogenous treatment with rhIL-33 is able to modulate apoptosis, Table S1: Primer pairs used for cDNA amplification of genes differentially expressed in the two cell lines.
