# Supplementary material for: Proinflammatory Interleukin-33 Induces Dichotomic Effects on Cell Proliferation in Normal Gastric Epithelium and Gastric Cancer
Source: Int J Mol Sci. 2021 May 28;22(11):5792. doi: 10.3390/ijms22115792 (PMC8197967; doi:10.3390/ijms22115792)
Supplement: Supplementary file 1 [file ijms-22-05792-s001.zip › ijms-1155016-supplementary.pdf]

International Journal of *Molecular Sciences*

MDPI

Article

Proinflammatory Interlekin-33 Induces Dichotomic Effects on Cell Proliferation in Normal Gastric Epithelium and Gastric Cancer

Laura Francesca Pisani [1], Gian Eugenio Tontini [2,3], Carmine Gentile [2], Beatrice Marinoni [2], Isabella Teani [2], Nicoletta Nandi [2], Pasquale Creo [1], Emanuele Asti [1,2], Luigi Bonavina [1,2], Maurizio Vecchi [3,4] and Luca Pastorelli [5,6,*]

[1] Gastroenterology ans Endoscopy Unit, IRCCS Policlinico San Donato, San Donato Milanese (Italy)
[2] Department of Biomedical Science for Health, Università degli Studi di Milano, Milano (Italy)
[3] Gastroenterology and Endoscopy Unit, Foundation IRCCS Ca' Granda Ospedale Maggiore Policlinico, Milan, Italy.
[4] Department of Pathophysiology and Trasplantation, Università degli Studi di Milano, Milano (Italy)
[5] Gastroenterology and Liver Unit, ASST Santi Paolo e Carlo, Ospedale San Paolo, Milano (Italy)
[6] Department of Health Sciences, Università degli Studi di Milano, Milano (Italy)
[7] Division of General Surgery, IRCCS Policlinico San Donato, San Donato Milanese (Italy)
* Corresponding author: Luca Pastorelli, MD, PhD ASST Santi Paolo e Carlo Via Di Rudinì, 8 - Milano (Italy) Phone: +39 0252774683 e-mail: luca.pastorelli@unimi.it

Supplementary Materials and Methods

Protein extraction and Western blot on cell lines

Proteins were extracted from cells using TRIzol® reagent (Life Technologies, Monza, Italy), following the manufacturer's guidelines, and suspended in sterile water containing protease and phosphatase inhibitors (Sigma-Aldrich, Milan, Italy). Briefly, proteins were isolated from the phenol–ethanol supernatant layer using protein precipitation. For protein precipitation, 1.5 mL of isopropanol was added to the phenol–ethanol supernatant and incubated for 10 minutes at room temperature, centrifuged at 12.000× *g* for 10 minutes at 4°C, and the supernatant removed. Then, the protein pellet was washed with 0.3 M guanidine hydrochloride in 95% ethanol. Finally, the protein pellet was air-dried for 5–10 minutes, and resuspended in 20 µL of 1% SDS and incubated at 50 °C in a heat block until the resuspension was completed. Then, 30 µg of proteins was loaded on SDS-PAGE electrophoretic gel (10%), followed by a nitrocellulose membrane-blot and tested with mouse monoclonal anti-IL-33 antibody 1:2000, ON, 4 °C (MABF204; Merk-Millipore, Milan, Italy) and rabbit polyclonal anti-IL-33 R ST2 antibody 1:2000, ON, 4 °C (06-1116; Merk-Millipore, Milan, Italy). Rabbit monoclonal anti-calnexin antibody [EPR3632] (ab92573) (Abcam, Milan, Italy) 1:20000, ON, 4 °C was used as a housekeeping protein. Horseradish peroxidase-conjugated (HRP) goat anti-mouse (AP308 P) (Merk-Millipore, Milan, Italy) and goat anti-rabbit (AP187 P) were used as secondary antibodies at a dilution of 1:5000 for 2 hours.

Table S1. Primer pairs used for cDNA amplification of genes differentially expressed in the two cell lines.

Gene	Forward	Revers
human IL-33	GACTCCTCCGAACACAGAGC	TGCTTGCTGTGTTCTTCCAC
human ST2 L	TTGCAAGGACAGCATCAAAG	GTCTGTGTTCCTGCCCAAAT
human CASP3	ACATCTCGGTCTGGTACAG	ACATCACGCATCAATTCCAC
human KPNA2	GTTGGCTCTCCTTGCAGTTC	GGGGTGCAGGATTCTTGTTG
human BCCIP	GAACTCGCGGTCACAGTACT	CACATGCGCTGATCAGTAGG
human CCNC	CTAGCCCAGCCTAGCAGAAA	ACTTCATGTAGTTTGCCCAGC
human CCNE1	CTCTTCTGTCTGTTGCAGCG	TTGCCCTGTTTGATGCCATC
human CCNB1	GGTTGTTGCAGGAGACCATG	AACATGGCAGTGACACCAAC
human GAPDH	CCATCACCATCTTCCAGGAG	CCTGCTTCACCACCTTCTTG
human S14	GTGTGACTGGTGGGATGAAGG	TTGATGTGTAGGGCGGTGATAC

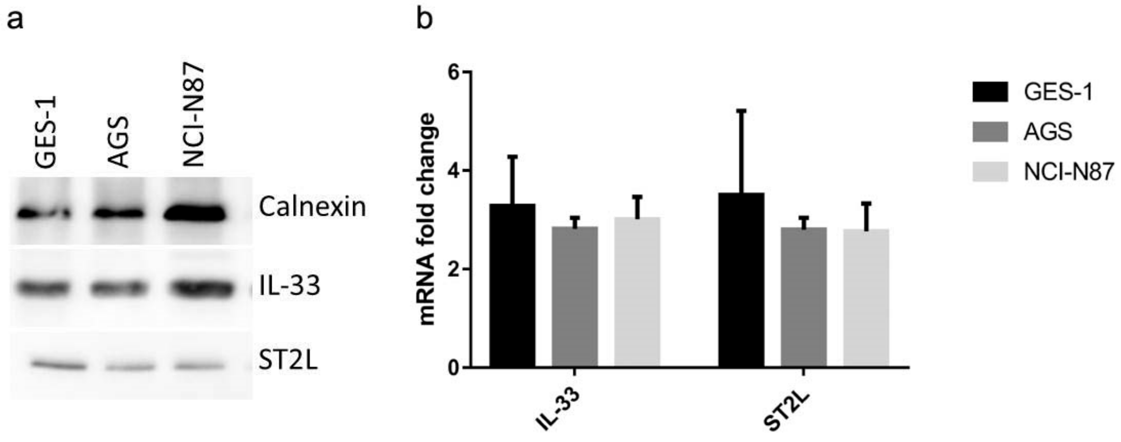

Figure S1. IL-33 and its receptor ST2 proteins, detected (a) by Western blot in the three cell lines and mRNA expression (b) by real-time PCR, show that IL-33 and ST2 L were present in GES-1, AGS, and NCI-N87 cell lines. Calnexin antibody was used as a reference control protein. RealTime PCR data are shown as mean ± SD.

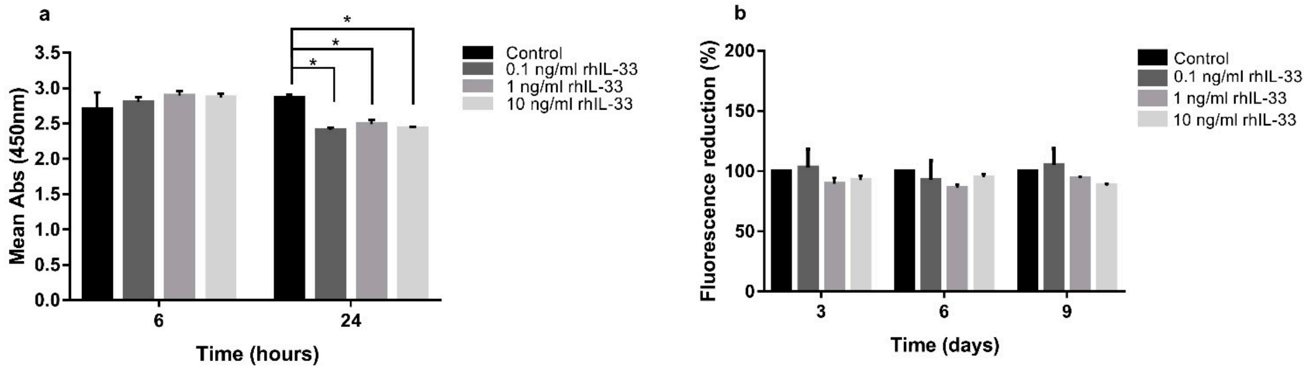

Figure S2. Exogenous administration of rhIL-33 reduced the activation of the proliferative metabolic pathway in neoplastic gastric epithelial cells, while the same challenge on neoplastic cells did not activate proliferation. Colorimetric XTT assay showed the antiproliferative effect on NCI-N87 (a) after 24 h treatment, and fluorescent CFSE assay (b) did not show any statically significant variation in proliferation. Data are shown as mean ± SD. * $p < 0.05$.

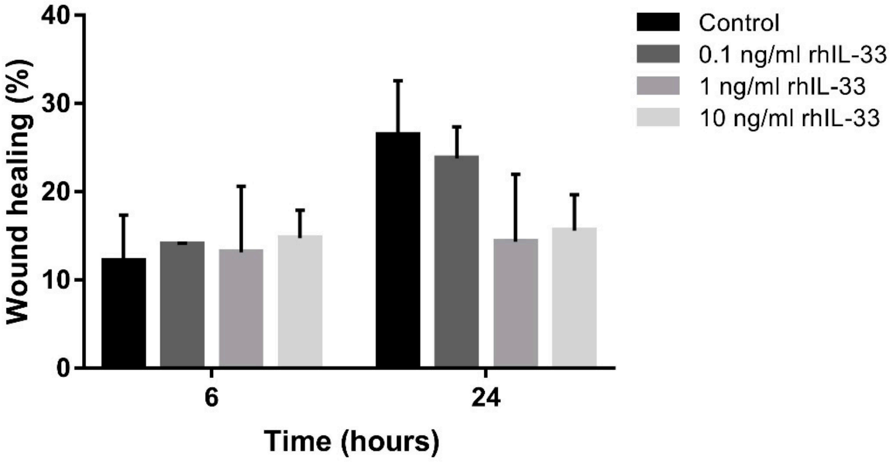

Figure S3. Administration of rhIL-33 for 6 and 24 h migration delayed the wound closure in neoplastic cells NCI-N87 after 24 h treatment. Data are shown as mean ± SD. * $p < 0.05$.

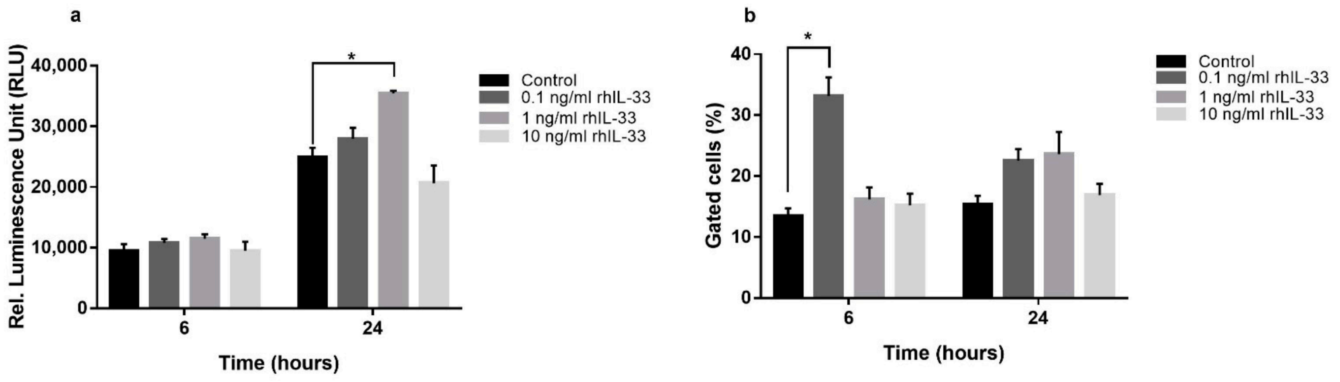

Figure S4. Exogenous treatment with rhIL-33 is able to modulate apoptosis. Quantitation of relative luminescence unit (RLU) for caspase 3/7 activity and the percentage of annexin-V-positive cells by cytofluorimetric assay showed an increase in both (a) early apoptosis and (b) activation of executioner caspases in the NCI-N87 cell line. Data are shown as mean ± SD. * $p < 0.05$.
